# Pregnancy and Neonatal Outcomes Among Women With Intellectual and Developmental Disabilities: A Systematic and Literature Review

**DOI:** 10.7759/cureus.114346

**Published:** 2026-08-11

**Authors:** Saadia Hassan, Shahela Nasir, Shahnawaz Pathan, Taharunnisa Syed, Arindam Ghosh, Javeria Gul, Areej Hamid, Nudrat Farid, Mariam Faiz Shakouhi, Lana Omar Mohammad Saleh, Muhammad Naveed Ullah Khan

**Affiliations:** 1 Obstetrics and Gynecology, Shaikh Zayed Medical College and Hospital, Rahim Yar Khan, PAK; 2 Obstetrics and Gynecology, Frimley Park Hospital, Surrey, GBR; 3 Obstetrics and Gynecology, Rawalpindi Medical University, Rawalpindi, PAK; 4 Obstetrics and Gynecology, Manchester University Hospitals NHS Foundation Trust, Manchester, GBR; 5 Pediatrics, Al-Ameen Medical College, karnataka, IND; 6 Pediatrics, Pilgrim Hospital, Lincolnshire, GBR; 7 Obstetrics and Gynecology, King Salman Specialized Hospital, Taif, SAU; 8 Biochemistry, Dr. B.C. Roy Multispeciality Medical Research Centre, Kharagpur, IND; 9 Obstetrics and Gynecology, Khyber Medical College, Peshawar, PAK; 10 General Practice, Sunderland Royal Hospital, Sunderland, GBR; 11 Obstetrics and Gynecology, Foundation University Medical College, Rawalpindi, PAK; 12 Medicine, Fazaia Medical College, Islamabad, PAK; 13 Obstetrics and Gynecology, Azerbaijan Medical University, Baku, AZE; 14 Obstetrics and Gynecology, Prince Hamza Hospital, Amman, JOR; 15 Inpatient General Adult Psychiatry, Rehman Medical College, Peshawar, PAK; 16 Inpatient General Adult Psychiatry, South West London and St George's Mental Health NHS Trust, London, GBR

**Keywords:** developmental disabilities, intellectual disabilities, literature review, neonatal outcomes, substantial health

## Abstract

Substantial health and social inequalities exist among women with intellectual and developmental disabilities (IDD), which can have a negative impact on maternal and neonatal health. For this review, an extensive search of the databases PubMed, Embase, Scopus, and Cochrane Library was performed from their inception to June 2026. Retrospective studies on outcomes of mothers and babies with IDD were included if they met the inclusion criteria. The quality was evaluated with the Risk of Bias In Non-randomized Studies of Interventions (ROBINS-I) tool. Retrospective cohort studies comprising more than 8.4 million pregnancies were included. Compared with women without IDD, women with IDD had higher rates of preterm birth (7.4%-2 3.3% vs. 5.0%-11.6%), small for gestational age (SGA) infants (13.1%-17.0%), low birth weight (LBW) (15.1%-15.4%), and neonatal intensive care unit (NICU) admission (13.3%-20.6% vs. 5.0%-11.7%). Adverse neonatal outcomes were also more common, including stillbirth (aOR=2.40, 95% CI 1.70-3.40), neonatal death (aRR=2.31, 95% CI 1.38-3.87), and infant mortality (PR=4.93, 95% CI 3.73-6.43). Maternal IDD was associated with severe maternal morbidity (aRR=1.61-4.82) and maternal mortality (aRR=11.19, 95% CI 2.40-52.19). The results indicate a need to provide targeted prenatal care and multidisciplinary interventions to improve the outcomes of women with IDD and their infants.

## Introduction and background

Intellectual and developmental disabilities (IDD) are a group of neurodevelopmental conditions that involve the brain, occur during the developmental period, and are characterized by significant limitations in intellectual functioning and adaptive behavior, communication, learning, and social skills [1]. IDD is used to refer to intellectual disability and other developmental disabilities, which may have different clinical presentations, functional impairments, and health service requirements, with an estimated prevalence of 1-3% of the population worldwide, and many of those with IDD also having functional limitations and health service needs as adults [2,3]. Better care, education and community support services have contributed to better survival and life expectancy for individuals with IDD, and an increasing number have reached reproductive age and become pregnant [4].

Pregnancy among women with IDD has increasingly been recognized as an important public health concern. While there have been challenges in accessing family planning and parenthood for people with IDD in the past, more recent evidence shows that many women with IDD have a desire to and experience pregnancy. This group of women is also likely to experience significant health disparities, including a higher prevalence of chronic medical and mental health conditions than women without disabilities, socioeconomic disadvantage, and barriers to prenatal and obstetric care [5] that vary depending on the definition of IDD used, methods of case identification, healthcare setting, and population characteristics [6]. These differences can increase the risk for adverse maternal and fetal outcomes. Findings from previous studies have been inconsistent about the magnitude of risk for various outcomes, including cesarean delivery, preterm birth and neonatal complications, and perinatal mortality [6,7]. Such variations could be attributed to variation in study design, population characteristics, definition of IDD, and adjustment for maternal comorbidities and socioeconomic factors.

While there have been previous studies investigating pregnancy outcomes for women with disabilities, most have included multiple disability groups or only analyzed specific maternal outcomes without reporting on the outcomes of neonates and infants specifically for women with IDD. In addition, several large observational studies have been published in recent years, highlighting the need for an updated systematic synthesis of the available evidence. [8] Previous reviews, including those focusing on women with disabilities broadly, have not exclusively evaluated women with IDD or have combined multiple disability categories, limiting the ability to determine IDD-specific risks. Therefore, this review provides an updated synthesis focusing specifically on women with IDD and evaluates maternal, obstetric, neonatal, infant, healthcare, and social outcomes.

This systematic review aimed to synthesize observational evidence comparing maternal, obstetric, neonatal, and infant outcomes among pregnant women with intellectual and developmental disabilities and women without these disabilities. Eligible studies included observational designs, and outcomes of interest included maternal pregnancy complications, delivery outcomes, neonatal morbidity, and infant outcomes. The findings were synthesized through narrative analysis and quantitative meta-analysis where appropriate.

## Review

Methodology

Study Design And Protocol Registration

This systematic review followed the Preferred Reporting Items for Systematic Reviews and Meta-Analyses (PRISMA) 2020 guidelines to ensure complete reporting in a transparent and methodologically sound manner [8]. The review protocol has been registered in the International Prospective Register of Systematic Reviews (PROSPERO) (Registration No. CRD420261433319). Cochrane Handbook for Systematic Reviews of Interventions (version 6.5) recommendations were followed for methodological procedures to conduct evidence synthesis.

Search Strategy

A comprehensive literature search was conducted in PubMed, Embase, Scopus, and the Cochrane Library from database inception to June 30, 2026. The search included controlled vocabulary terms (MeSH/Emtree) and free-text terms related to intellectual and developmental disabilities, pregnancy, and maternal and neonatal outcomes. Reference lists of relevant reviews and included studies were also manually screened to identify additional eligible studies. Retrieved records were imported into reference management software, duplicates were removed, and titles, abstracts, and full texts were independently screened by two reviewers according to predefined eligibility criteria. All databases were searched using detailed search strategies as detailed in Table 1. All identified records were imported into reference management software, and duplicate records were eliminated. Titles and abstracts were independently screened for eligibility by two reviewers. The full-text of potentially relevant articles was reviewed. 

**Table 1:** Detailed electronic search strategy used across databases

PubMed, n=2114	Embase =20	Scopus n=995	Cochrane n=211
("Intellectual Disability"[MeSH Terms] OR "Developmental Disabilities"[MeSH Terms] OR "Intellectual Disability"[All Fields] OR "developmental disability"[All Fields] OR "Developmental Disabilities"[All Fields] OR "intellectual and developmental disabilities"[All Fields] OR ("intellect dev disabil"[Journal] OR "idd"[All Fields])) AND ("Pregnancy"[MeSH Terms] OR "Pregnancy Outcome"[MeSH Terms] OR ("Pregnancy"[MeSH Terms] OR "Pregnancy"[All Fields] OR "pregnancies"[All Fields] OR "pregnancy s"[All Fields]) OR ("pregnant"[All Fields] OR "pregnants"[All Fields]) OR ("maternally"[All Fields] OR "maternities"[All Fields] OR "maternity"[All Fields] OR "mothers"[MeSH Terms] OR "mothers"[All Fields] OR "maternal"[All Fields]) OR ("obstetric"[All Fields] OR "obstetrically"[All Fields] OR "obstetrics"[MeSH Terms] OR "obstetrics"[All Fields] OR "obstetrical"[All Fields])) AND ("Premature Birth"[MeSH Terms] OR "infant, premature"[MeSH Terms] OR "infant, low birth weight"[MeSH Terms] OR "infant, small for gestational age"[MeSH Terms] OR "intensive care units, neonatal"[MeSH Terms] OR "Infant Mortality"[MeSH Terms] OR "Perinatal Mortality"[MeSH Terms] OR "Stillbirth"[MeSH Terms] OR "Maternal Mortality"[MeSH Terms] OR "Maternal Health"[MeSH Terms] OR "preterm birth"[All Fields] OR ("Premature Birth"[MeSH Terms] OR ("premature"[All Fields] AND "birth"[All Fields]) OR "Premature Birth"[All Fields]) OR "low birth weight"[All Fields] OR "LBW"[All Fields] OR "small for gestational age"[All Fields] OR "SGA"[All Fields] OR ("intensive care units, neonatal"[MeSH Terms] OR ("intensive"[All Fields] AND "care"[All Fields] AND "units"[All Fields] AND "neonatal"[All Fields]) OR "neonatal intensive care units"[All Fields] OR "nicu"[All Fields]) OR ("Stillbirth"[MeSH Terms] OR "Stillbirth"[All Fields] OR "stillbirths"[All Fields]) OR "neonatal mortality"[All Fields] OR "Infant Mortality"[All Fields] OR "maternal morbidity"[All Fields] OR "Maternal Mortality"[All Fields])	('intellectual disabilities'/exp OR 'intellectual disabilities' OR (intellectual AND disabilities)) AND ('developmental disabilities'/exp OR 'developmental disabilities' OR (developmental AND disabilities)) AND ('maternal outcomes'/exp OR 'maternal outcomes' OR (('maternal'/exp OR maternal) AND ('outcomes'/exp OR outcomes))) AND ('neonatal outcomes'/exp OR 'neonatal outcomes' OR (neonatal AND ('outcomes'/exp OR outcomes)))	TITLE-ABS-KEY ( ( "intellectual disability" OR "developmental disabilities" OR "intellectual and developmental disabilities" OR IDD ) AND ( pregnan* OR matern* ) AND ( "preterm birth" OR "low birth weight" OR "small for gestational age" OR stillbirth OR "neonatal mortality" OR "infant mortality" OR NICU ) )	#1 MeSH descriptor: [Intellectual Disability] explode all trees #2 MeSH descriptor: [Developmental Disabilities] explode all trees #3 ("intellectual disability" OR "developmental disability" OR "developmental disabilities" OR "intellectual and developmental disabilities" OR IDD):ti,ab,kw #4 #1 OR #2 OR #3 #5 MeSH descriptor: [Pregnancy] explode all trees #6 MeSH descriptor: [Pregnancy Outcome] explode all trees #7 MeSH descriptor: [Pregnant Women] explode all trees #8 (pregnan* OR matern* OR obstetric*):ti,ab,kw #9 #5 OR #6 OR #7 OR #8 #10 MeSH descriptor: [Premature Birth] explode all trees #11 MeSH descriptor: [Infant, Low Birth Weight] explode all trees #12 MeSH descriptor: [Infant, Small for Gestational Age] explode all trees #13 MeSH descriptor: [Intensive Care Units, Neonatal] explode all trees #14 MeSH descriptor: [Infant Mortality] explode all trees #15 MeSH descriptor: [Stillbirth] explode all trees #16 MeSH descriptor: [Maternal Mortality] explode all trees #17 ("preterm birth" OR prematur* OR "low birth weight" OR LBW OR "small for gestational age" OR SGA OR NICU OR stillbirth* OR "neonatal mortality" OR "infant mortality" OR "maternal morbidity" OR "maternal mortality"):ti,ab,kw #18 #10 OR #11 OR #12 OR #13 OR #14 OR #15 OR #16 OR #17 #19 #4 AND #9 AND #18

Eligibility Criteria

Studies were selected using predefined eligibility criteria related to population, exposure, outcomes, study design and publication characteristics. Eligible studies were those that reported maternal, obstetric, neonatal or infant outcomes and included pregnant women with intellectual and developmental disabilities (IDD). Research is needed to have a comparison group of women without IDD and adequate outcome data for women with IDD in Table 2.

**Table 2:** Eligibility criteria for study selection IDD - intellectual and developmental disabilities; NICU - neonatal intensive care unit

Domain	Inclusion criteria	Exclusion criteria
Population	Pregnant women with IDD	Studies involving non-pregnant populations or populations without IDD
Exposure	Diagnosis of intellectual disability, developmental disability, or intellectual and developmental disabilities	Studies not specifically evaluating IDD
Outcomes	Maternal, obstetric, neonatal, or infant outcomes including preterm birth, low birth weight, small for gestational age, NICU admission, mortality, maternal morbidity, and related outcomes	Studies not reporting relevant pregnancy or neonatal outcomes
Study design	Cohort studies, case-control studies, cross-sectional studies, and population-based observational studies	Case reports, case series, editorials, conference abstracts, narrative reviews, systematic reviews, and animal studies
Publication characteristics	Peer-reviewed full-text studies published in English	Non-English studies, duplicate publications, and studies lacking sufficient outcome data

Data Extraction

Data was independently extracted by two reviewers using a standardized Excel sheet (Microsoft, Redmond, Washington). Characteristics (author, year, country, study design), participant characteristics (sample size, maternal age, race/ethnicity, socioeconomic indicators, body mass index, and comorbidities), and reported maternal and neonatal outcomes were extracted. Neonatal outcomes included preterm birth, low birth weight, small for gestational age (SGA), admission to neonatal intensive care unit (NICU), neonatal morbidity, stillbirth, neonatal mortality, and infant mortality. 

When there were discrepancies in data extraction, we discussed them and used a third reviewer to reconcile them. 

Quality Assessment

The Risk of Bias In Non-randomized Studies of Interventions (ROBINS-I) tool was considered for studies [9]. Any disagreements in the quality assessment were resolved through discussion and consensus with a third reviewer. 

Results

Study Selection Process

**Figure 1:**
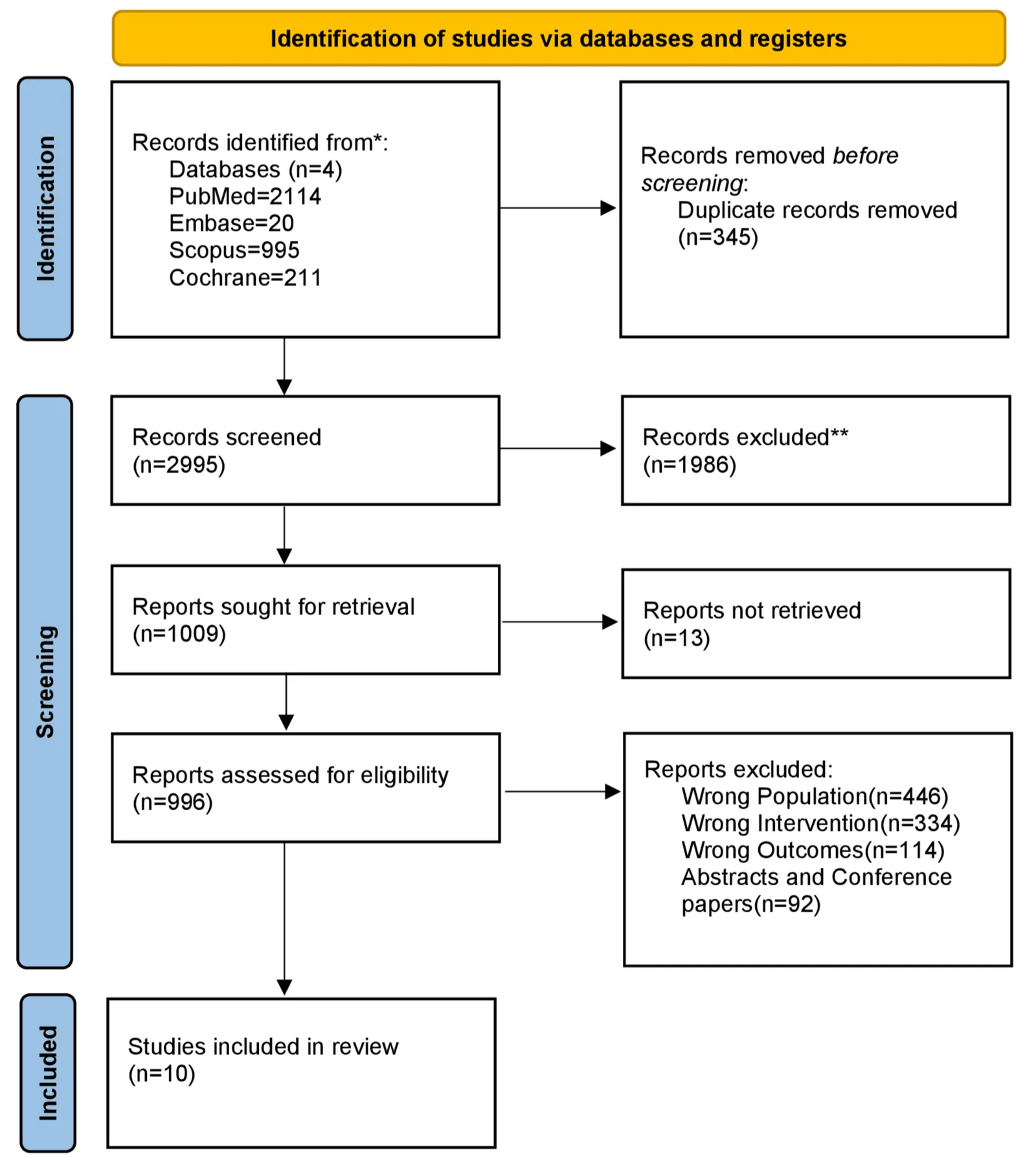
PRISMA 2020 flow diagram of study selection process PRISMA 2020 flow diagram illustrating the study selection process, including identification, screening, eligibility assessment, and final inclusion of studies in the systematic review. * Records identified from electronic databases.  ** Records excluded during title and abstract screening.

Database searching (PubMed, n=2114; Embase, n=20; Scopus, n=995; Cochrane, n=211) yielded a total of 3340 records. The titles and abstracts of 2995 studies were screened, with 1986 excluded as duplicates. The full texts of 1009 articles were sought for retrieval, and 996 studies were assessed for eligibility after excluding 13 unretrieved reports. After full-text screening, 986 articles were excluded for not meeting the inclusion criteria: irrelevant population (n=446), irrelevant interventions/exposures (n=334), irrelevant outcomes (n=114), irrelevant publication type (abstracts/conference papers; n=92), leaving 10 studies for the final systematic review as shown in Figure 1.

Risk of Bias Assessment Using ROBIN-I

**Figure 2:**
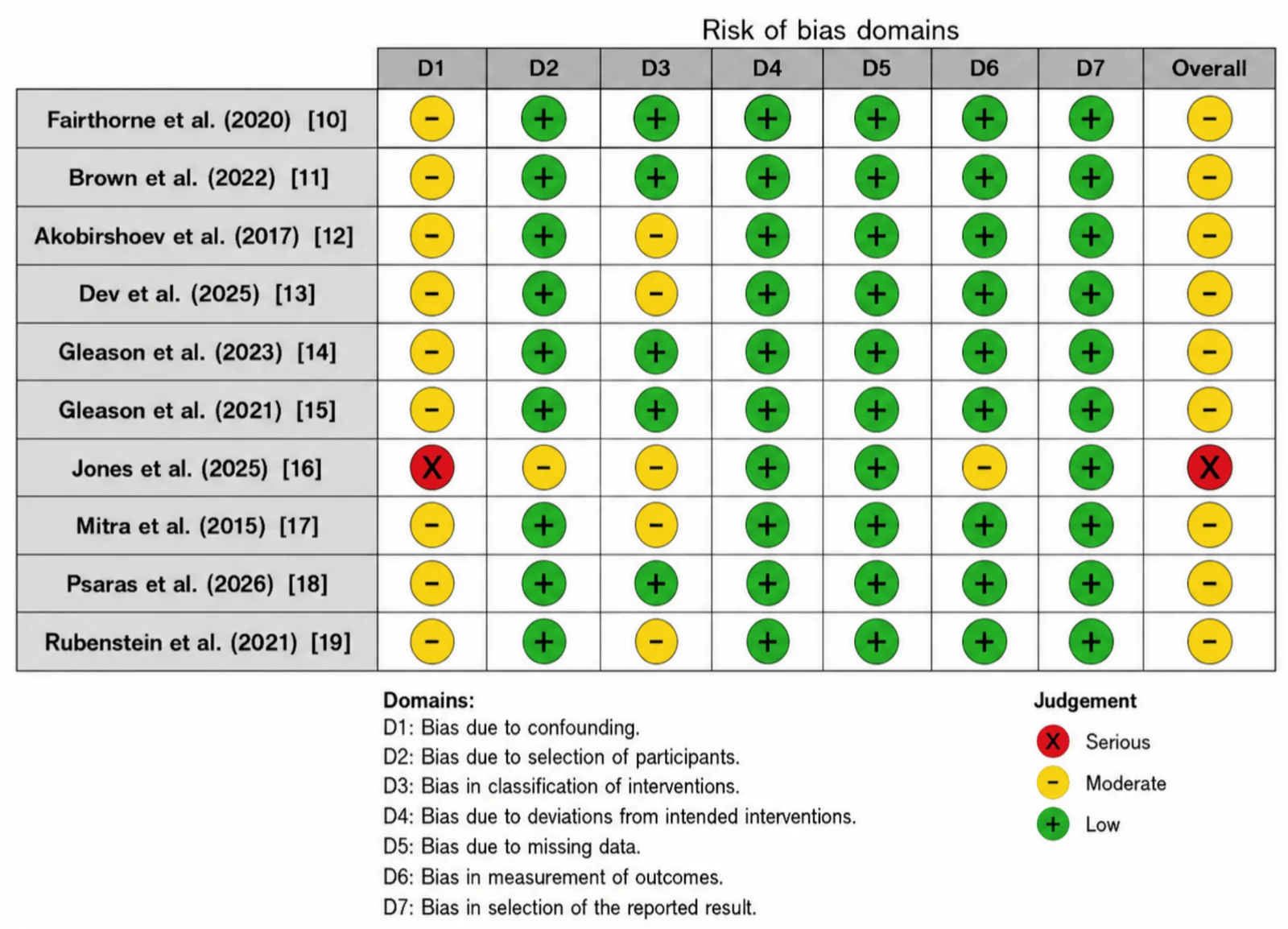
Traffic light plot of risk of bias domains using ROBINS-I for the 10 included studies Traffic light plot showing the risk of bias assessment for each included study across the seven ROBINS-I domains and the overall risk of bias judgment. ROBINS-I - Risk of Bias In Non-randomized Studies of Interventions

**Figure 3:**
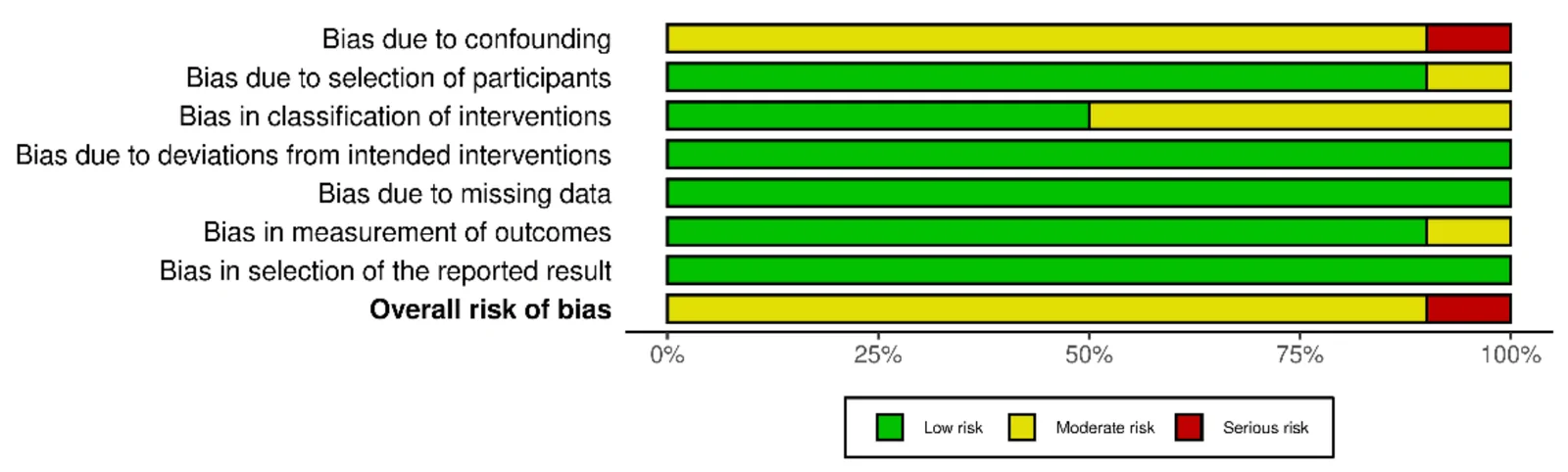
Traffic light plot summary for included studies using ROBIN-I of included 10 studies Summary traffic light plot illustrating the proportion of studies rated as low, moderate, or serious risk of bias across each ROBINS-I domain and overall risk of bias. ROBINS-I - Risk of Bias In Non-randomized Studies of Interventions

The main methodological concern was confounding bias (D1), with nine studies at moderate risk of bias and one at serious risk. The selection of participants (D2) was generally low; nine studies were at low risk, and one was at moderate risk. The classification of exposure (D3) was assessed as low risk in six studies and moderate risk in four studies due to potential misclassification from administrative coding of IDD. Bias arising from deviations from intended exposure (D4), missing data (D5), and selection of reported results (D7) was uniformly rated as low risk for all included studies. The risk of bias in outcome measurement (D6) was mostly low, and only one study had a moderate risk. Overall, nine studies were rated as having a moderate risk of bias, and one study was judged to have a serious overall risk of bias, mainly due to concerns about confounding and exposure classification (Figure 2, Figure 3).

Baseline Characteristics

More than 8.4 million pregnancies were included in the ten studies [10-19], which took place in the US, Canada, and Australia. The women with IDD tended to be younger than the controls, with mean ages between 25.3 and 29.0 years, and 37.1-48.3 % below the age of 25 years. White ethnicity was the predominant racial group (35.0%-70.0%), while Black (19.0%-22.9%), Hispanic (16.8%-41.0%), and Aboriginal women (22.5%) were also represented. Socioeconomic disadvantage was prevalent, with 27.0%-32.2% lacking a high school education, 27.5%-37.6% in the lowest-income groups, and 12.6%-79.6% on public insurance. The proportion of respondents who were married/partnered ranged from 28.6% to 72.1%. The incidence of obesity was reported as 27.6% of women, with 52% overweight or obese. Women with IDD had consistently higher smoking during pregnancy (29.7%) and physical and mental health comorbidities (Table 3).

**Table 3:** Characteristics of included studies and baseline demographic and clinical characteristics of mothers with intellectual and developmental disabilities Values are presented as mean ± standard deviation (SD), median, percentage, or proportion as reported in the original studies. Comorbidities include pre-pregnancy physical, psychiatric, and behavioral health conditions where available. ADHD - attention-deficit/hyperactivity disorder; BMI - body mass index; HCUP-NIS - Healthcare Cost and Utilization Project Nationwide Inpatient Sample; ICES - Institute for Clinical Evaluative Sciences; IDEA - Intellectual Disability Exploring Answers Database; ID - intellectual disability; IDD - intellectual and developmental disabilities; IF - intellectual functioning; NR - not reported; SES - socioeconomic status; SOMI - Statewide Opportunity for Maternal and Infant Health Database

Study (author, year)	Country / data source	Study design	Sample size (IDD / control)	Maternal age (IDD vs control)	Race / ethnicity	Socioeconomic status / insurance	Marital status	BMI / obesity	Comorbidities / baseline characteristics
Fairthorne et al. (2020) [10]	Western Australia, Australia (IDEA Database)	Retrospective Cohort	943 / 7,800	60.7% aged 20-29 years	22.5% Aboriginal	27.5% in lowest SES quintile	46.2% partnered	NR	Smoking during pregnancy: 29.7%; low SES more common
Brown et al. (2022)[11]	Ontario, Canada (ICES Health Administrative Databases)	Retrospective Cohort	2,207 / 1,593,354	45.4% aged <25 years	Not reported	37.6% in lowest income quintile	NR	NR	Depression/anxiety more common among women with IDD
Akobirshoev et al. (2017)[12]	United States (HCUP Nationwide Inpatient Sample)	Retrospective Cohort	1,897 / 4,194,938	25.9 vs 27.6 years	47.2% White, 19.6% Black	57.0% Medicaid; 16.4% Medicare	NR	NR	Higher burden of chronic physical conditions
Dev et al. (2025)[13]	Texas, USA (Medicaid Claims Database)	Retrospective Cohort	26,017 / 895,201	25.3 ± 5.8 years	64.5% White, 21.9% Black	32.2% less than high school education	32.0% married	NR	High overall comorbidity burden; pre-existing hypertension and diabetes more common
Gleason et al. (2023)[14]	United States (Consortium on Safe Labor)	Retrospective Cohort	2,142 / 221,311	28.8 ± 6.4 vs 27.6 ± 6.2 years	62.4% White, 19.0% Black	38.9% public insurance/self-pay	54.8% married	Mean BMI 26.3 kg/m² vs 25.4 kg/m²	Higher prevalence of obesity and chronic medical conditions
Gleason et al. (2021) [15]	United States (Consortium on Safe Labor)	Retrospective Cohort	2,074 / 221,311	28.8 ± 6.4 years	62.4% White, 19.0% Black	38.9% public insurance/self-pay	54.8% married	Mean BMI 26.3 kg/m² vs 25.4 kg/m²	Higher prevalence of chronic physical and mental health conditions
Jones et al. (2025)[16]	Utah, USA (Utah Population Database)	Retrospective Cohort	2,250 IDD mothers	26.6 ± 5.8 years	56.4% White, 38.8% Hispanic	12.6% Medicaid coverage	72.1% married	27.6% obesity (BMI ≥30 kg/m²)	Maternal infection reported in 8.4%; obesity common
Mitra et al. (2015)[17]	Massachusetts, USA (PELL Database)	Retrospective Cohort	703 / 865,369	37.1% aged <25 years	65.2% White, 16.8% Hispanic	79.6% public insurance	34.4% married	NR	Greater social vulnerability; lower paternity establishment
Psaras et al. (2026)[18]	California, USA (SOMI Database)	Retrospective Cohort	4,492 / 134,760	29 ± 7 vs 30 ± 6 years	41.0% Hispanic, 35.0% White	59.0% public insurance	Not reported	52% overweight/obese (BMI ≥25 kg/m²)	Lower adequacy of prenatal care; obesity common
Rubenstein et al. (2021) [19]	Wisconsin, USA (Medicaid Linkage Database)	Retrospective Cohort	1,696 / 265,699	48.3% aged <25 years	70.0% White, 22.9% Black	27.0% less than high school education	28.6% married	NR	Higher social disadvantage and healthcare needs

Systematic Review of Outcomes

The outcomes are mentioned in Table 4. The Table presents the reported effect estimates for adverse pregnancy, maternal, neonatal, and infant outcomes comparing women with IDD and control populations across included studies.

**Table 4:** Summary of maternal and neonatal outcomes reported in included studies PTB - preterm birth; LBW - low birth weight; SGA - small for gestational age; NICU - neonatal intensive care unit; SMM - severe maternal morbidity; RR - risk ratio; aRR - adjusted risk ratio; aRRR - adjusted relative risk ratio; aOR - adjusted odds ratio; PR - prevalence ratio; NR - not reported

Study (author, year)	Sample size (IDD/control)	PTB	LBW	SGA	NICU admission	Stillbirth	Neonatal death	Infant death	SMM	Maternal death	Cesarean delivery	Apgar <5
Fairthorne et al. (2020) [10]	943 / 7800	aRRR 1.24 (0.84-1.84)	aRRR 1.18 (0.87–1.61)	NR	NR	aRRR 0.86 (0.25-2.93)	NR	NR	NR	NR	NR	NR
Brown et al. (2022) [11]	2207 / 1,593,354	aRR 1.37 (1.19-1.58)	NR	aRR 1.37 (1.24-1.51)	aRR 1.53 (1.40-1.67)	NR	NR	NR	NR	NR	NR	NR
Akobirshoev et al. (2017) [12]	1897 / 4,194,938	aOR 1.46 (1.26-1.69)	aOR 1.61 (1.27-2.05)	NR	NR	aOR 2.40 (1.70-3.40)	NR	NR	NR	NR	NR	NR
Dev et al. (2025) [13]	26,017 / 895,201	aRR 1.57 (1.53-1.62)	aRR 1.64 (1.60-1.69)	NR	NR	NR	NR	NR	aRR 4.12 (3.79-4.47)	NR	NR	NR
Gleason et al. (2023) [14]	2142 / 221,311	aRR 1.77 (1.62-1.94)	NR	aRR 1.25 (1.11-1.41)	aRR 1.70 (1.54-1.87)	NR	aRR 2.31 (1.38-3.87)	NR	NR	NR	NR	NR
Gleason et al. (2021) [15]	2074 / 221,311	NR	NR	NR	NR	NR	NR	NR	aRR 1.61 (1.46-1.76)	aRR 11.19 (2.40-52.19)	aRR 1.33 (1.25-1.42)	NR
Jones et al. (2025) [16]	2250 (IDD only)	14.2%	11.8%	NR	4.8%	NR	NR	NR	NR	NR	24.8%	NR
Mitra et al. (2015) [17]	703 / 865,369	RR 1.70 (1.50-2.00)	RR 2.20 (2.00-2.50)	NR	NR	NR	NR	NR	NR	NR	NR	RR 3.40 (2.40-4.80)
Psaras et al. (2026) [18]	4492 / 134,760	aRR 2.34 (2.18-2.51)	NR	aRR 1.56 (1.44-1.68)	aRR 2.76 (2.56-2.98)	NR	NR	NR	NR	NR	NR*	NR
Rubenstein et al. (2021) [19]	1696 / 265,699	PR 1.64 (1.42-1.89)	NR	PR 1.42 (1.25-1.61)	PR 1.70 (1.43-2.03)	NR	NR	PR 4.93 (3.73-6.43)	NR	NR	NR	NR

Preterm birth (<37 weeks): Eight studies [10,11-14,17-19] reported preterm birth. With reported effect estimates ranging from 1.24 to 2.34, moms with intellectual and developmental disabilities (IDD) generally had a significantly greater risk of preterm delivery than mothers without IDD. According to Fairthorne et al. (2020) [10], preterm birth occurred in 7.4% of non-Aboriginal women with IDD as opposed to 5.0% of controls (aRRR 1.24, 95% CI: 0.84-1.84). Preterm birth rates were 9.2% versus 6.2% (aRR 1.37, 95% CI: 1.19-1.58), according to Brown et al. (2022) [11]. According to Brown et al. (2022) [11], Akobirshoev et al. (2017) [12] and Dev et al. (2025) [13] found rates of 17.7% versus 10.4% (aRR 1.57, 95% CI: 1.53-1.62). Women with IDD had an estimated prevalence of roughly 13.0%, while controls had a prevalence of 8.0% (aOR 1.46, 95% CI: 1.26-1.69). While Gleason et al. (2023) [14] showed the highest prevalence at 23.3% compared with 11.6% among controls (aRR 1.77, 95% CI: 1.62-1.94), According to Mitra et al. (2015) [17], preterm birth occurred in 12.6% of pregnancies in women with IDD as opposed to 7.3% in controls (RR 1.70, 95% CI: 1.50-2.00). Psaras et al. (2026) [18] reported rates of 16.0% versus 7.0%, resulting in the greatest recorded increase in risk (aRR 2.34, 95% CI: 2.18-2.51). Rubenstein et al. (2021) [19] found that preterm birth occurred in 12.8% of pregnancies in women with IDD compared to 7.8% in controls (PR 1.64, 95% CI: 1.42-1.89). These data show a continuously higher risk of preterm birth among moms with IDD.

Fetal growth and birth weight outcomes: The relationship between fetal growth and birth weight outcomes. Fetal growth/birth weight outcomes. Four studies [11,14,18,19] reported small-for-gestational-age (SGA) babies. Brown et al. (2022) [11] reported SGA in 17.0% of infants born to mothers with IDD (aRR 1.37, 95% CI: 1.24-1.51). Gleason et al. (2023) [14] observed SGA in 13.1% of exposed infants (aRR 1.25, 95% CI: 1.11-1.41). Psaras et al. (2026) [18] reported SGA in 14.0% of infants (aRR 1.56, 95% CI: 1.44-1.68), while Rubenstein et al. (2021) [19] found SGA in 15.1% of exposed infants (PR 1.42, 95% CI: 1.25-1.61). In every study, maternal IDD was found to be associated with a higher risk of having an SGA infant.

Low birth weight (LBW) was reported in three studies [12,13,17]. The odds of LBW were significantly higher for mothers with IDD (aOR 1.61, 95% CI: 1.27-2.05) (Akobirshoev et al., 2017) [12]. Dev et al. (2025) [13] reported LBW in 15.4% of exposed infants (aRR 1.64, 95% CI: 1.60-1.69), while Mitra et al. (2015) [17] reported LBW in 15.1% of exposed infants and more than double the risk compared with controls (RR 2.20, 95% CI: 2.00-2.50). These findings suggest an increased risk of low birth weight among infants born to mothers with IDD, which is consistent across all mothers with IDD. One study reported low head circumference. Low head circumference was observed in 14.3% of infants born to mothers with IDD, and 10.7% of infants with controls, which results in a relative risk ratio of 1.24 (CI: 0.93-1.66).

Neonatal morbidity and intensive care utilization: Three studies [11,18,19] reported NICU admission. Psaras et al. (2026) [18] reported that the rates of NICU admission for infants exposed to maternal IDD were 14.0% compared to 5.0% for controls (aRR 2.76, 95% CI: 2.56-2.98). Brown et al. (2022) [11] reported NICU admission in 20.6% of exposed infants versus 11.7% of controls (aRR 1.53, 95% CI: 1.40-1.67). Rubenstein et al. (2021) [19] similarly found rates of 13.3% versus 7.9% (PR 1.70, 95% CI: 1.43-2.03). Overall, the infants of IDD mothers were more likely to need intensive neonatal care. 

Two studies [11,14] reported composite neonatal morbidity. For all three neonatal morbidity endpoints (sepsis, seizures, and respiratory complications), the authors of the Brown et al. (2022) [11] study found that the composite rate was higher in exposed infants than in control infants, with aRR ranging from 1.27 to 1.42 for all three outcomes and being 1.42 for the composite outcome (95% CI: 1.27-1.60). Gleason et al. (2023) [14] similarly reported higher rates of neonatal morbidity among exposed infants (24.1% vs. 13.7%; aRR 1.59, 95% CI: 1.45-1.74). One study reported respiratory distress syndrome. Gleason et al. (2023) [14] reported on 7.1% of infants with IDD and respiratory distress syndrome (RDS) compared to 3.2% for controls (aRR 2.02, 95% CI: 1.72-2.39). In one study, neonatal seizures were reported. Gleason et al. (2023) [14] observed neonatal seizures in 0.5% of exposed infants compared with 0.2% among controls (aRR 2.81, 95% CI: 1.54-5.14). Among mothers with IDD, 2.7% had infants with neonatal abstinence syndrome (NAS), compared to 0.48% of the control infants, aRR 1.53 (95% CI: 1.12-2.08). One study reported that infants were transferred to another hospital. Rubenstein et al. (2021) [19] reported transfer rates of 8.7% among exposed infants compared with 3.7% among controls (PR 2.38, 95% CI: 2.03-2.78).

Perinatal and infant mortality: Stillbirth was documented in two investigations. Fairthorne et al. (2020) [10] discovered stillbirth rates of 0.7% among mothers with IDD and 0.6% among controls (aRRR 0.86, 95% CI: 0.25-2.93). In contrast, Akobirshoev et al. (2017) [11] found a significantly higher risk of stillbirth among women with IDD (aOR 2.40, 95% CI: 1.70-3.40). One study documented a neonatal death. Gleason et al. (2023) [14] found that infants born to women with IDD had a significantly higher risk of neonatal death (aRR 2.31, 95% CI 1.38-3.87). One study reported infant mortality. Rubenstein et al. (2021) [19] discovered infant mortality within the first year of life in 3.2% of infants born to mothers with IDD compared to 0.7% in controls, indicating a fivefold increase in risk.

Maternal morbidity and mortality: There were two studies [13,15] reporting severe maternal morbidity (SMM). A group of women with IDD was found to have a significantly higher risk of SMM (aRR 4.82, 95% CI: 3.96-5.86) by Dev et al. (2025) [13]. A similar increase in severe maternal morbidity was also observed among women with disabilities overall by Gleason et al. (2021) [15] (aRR 1.61, 95% CI: 1.46-1.76). One study reported maternal mortality. The prevalence of maternal deaths was 0.09% for women with disabilities and 0.01% for controls (aRR 11.19; 95% CI: 2.40-52.19) (Gleason et al., 2021) [15]. A single study reported venous thromboembolism. Gleason et al. (2021) [15] observed venous thromboembolism in 1.3% of women with IDD compared with 0.18% among controls (aRR 6.08, 95% CI: 4.03-9.16). One study reported maternal septicemia or sepsis by Gleason et al. (2021) [15] found rates of 0.8% among women with IDD compared with 0.03% among controls (aRR 23.04, 95% CI: 13.01-40.57). One study by Gleason et al. (2021) [15] reported that blood transfusion rates were found to be 4.2% for women with IDD and 3.8% for controls (aRR 1.64, 95% CI: 1.27-2.12)

Obstetric complications and delivery outcomes: One study reported a premature rupture of membranes. Gleason et al. (2021) [15] found that women with IDD had a higher risk of PROM (aRR 1.16). One study reported preterm premature membrane rupture (PPROM) by Gleason et al. (2021) [15] observed a similar elevated risk of PPROM (aRR 1.55). Two studies [10,15] reported postpartum hemorrhage. Fairthorne et al. (2020) [10] found that 18.3% of women with IDD experienced postpartum hemorrhage compared to 14.4% of controls (RRR 1.33, 95% CI: 1.05-1.68). Gleason et al. (2021) [15] discovered rates of 7.1% and 6.6%, respectively (aRR 1.27, 95% CI: 1.08-1.49). Gleason et al. (2021) [15] found that 8.5% of women with IDD had surgical vaginal delivery, compared to 6.5% of controls (aRR 1.33, 95% CI: 1.27-1.39). Psaras et al. (2026) [18] discovered that women with IDD were more likely to have a cesarean birth, but procedures were less likely to be conducted for medical reasons (aRR 0.79, 95% CI: 0.70-0.89). Jones et al. (2025) [16] found that Medicaid coverage in an IDD cohort was linked with an increased risk of cesarean delivery (OR 1.76, 95% CI: 1.45-2.13). One research study reported a maternal infection. Jones et al. (2025) [16] discovered maternal infection in 8.4% of newborns to women with IDD. Akobirshoev et al. (2017) [12] reported on the length of hospital stays and found that women with IDD experienced considerably longer delivery-related hospitalization than women without IDD.

Postpartum, health behavior, and social outcomes: Two studies [17,19] reported breastfeeding at discharge. Among mothers with IDD, breastfeeding rates were 49.4%, while in the controls, 77.4% of mothers were breastfeeding. In the study by Rubenstein et al. (2021) [19], women with IDD were also less likely to initiate breastfeeding (57.8% vs. 69.9% (PR 0.82, 95% CI: 0.76-0.87)). In one study, smoking was reported during pregnancy. In a study by Fairthorne et al. (2020) [10], women with IDD were more likely to be smokers (29.7%) than controls (18.4%). Adequacy of prenatal care was reported in one study. Psaras et al. (2026) [18] found that 67% of prenatal care was adequate or better for women with IDD than for controls (72%). One of the studies reported paternity establishment. The results showed that women with IDD were generally found to be socially deprived, with 63.8% of them not naming a father on the birth certificate, contrasting with 33.4% of controls. 

In all of the studies included, maternal IDD was associated with poor neonatal, maternal, obstetric, and social outcomes. Women with IDD had an increased risk of low birth weight, small for gestational age, respiratory distress, neonatal seizures, stillbirth, neonatal death, infant mortality, severe maternal morbidity, maternal sepsis, venous thromboembolism, postpartum hemorrhage, operative delivery and cesarean birth. Furthermore, mothers with IDD were less likely to breastfeed, less likely to receive adequate prenatal care, and more likely to smoke during the pregnancy, to stay in the hospital longer, and to exhibit more social vulnerability than women without IDD.

Discussion

Summary of Findings

There is clear evidence from this systematic review that women with intellectual and developmental disabilities (IDD) have significantly worse maternal, obstetric, perinatal, and neonatal outcomes than non-IDD women. In the studies included, the risk for premature birth, small-for-gestational age (SGA) infants, low birth weight (LBW), neonatal intensive care unit (NICU) admission, neonatal morbidity, respiratory distress syndrome, neonatal seizures, stillbirth, neonatal death, infant mortality, severe maternal morbidity, postpartum hemorrhage, and cesarean delivery was all increased by maternal IDD, across studies. In addition, IDD women were less likely to start breastfeeding and more likely to have poor prenatal care, social vulnerability, and negative health behaviors during pregnancy. 

Comparison with Existing Literature

The results of these studies suggest that women with IDD make a unique segment of the obstetric population that must be better supported with multiple services and cared for with a focus on maternity. This review demonstrated the existing evidence that women with disabilities face severe inequities in reproductive health. Tarasoff et al. conducted a study on pregnancy, delivery and postpartum complications among women with disabilities, which showed an increased risk of pregnancy, delivery and postpartum complications, such as preterm births and cesarean delivery, compared with women without disabilities [20]. Likewise, another study by Tarasoff et al. showed that infants born to women with disabilities were more likely to be preterm, low birth weight, be admitted to the NICU, and have adverse neonatal outcomes [21].

Furthermore, the findings of fetal growth restriction and low birth weight are also corroborated by studies indicating that women with intellectual disabilities are at increased risk for adverse birth outcomes, perhaps because of the increased prevalence of chronic disease, socioeconomic disadvantage, and poor access to prenatal care [22,23]. Neonatal morbidity, respiratory complications and infant mortality rates were higher in the present review, and there is evidence that children born to mothers with disabilities experience significantly higher rates of adverse neonatal health outcomes [24,25]. 

Moreover, the increased risk of severe maternal morbidity, thromboembolic complications and maternal infections found in this review are consistent with more recent, population-based research showing that women with disabilities have significantly higher rates of serious pregnancy-related outcomes [26,27]. These differences might be due to medical comorbidities, access to health care, provider training, provider-patient communication, or social determinants of health; several authors have proposed these reasons [21,25]. Additionally, some previous literature has also identified ongoing problems women with intellectual disabilities experience in maternity care, such as lack of support, lack of access to health information, and lack of accommodations during pregnancy and postpartum [23,24]. 

Hence, these findings, when combined, collectively further underscore the systemic inequities faced by women with IDD across the reproductive continuum. 

Limitations and Strengths

There are certain limitations of our study. Firstly, most of the studies included were retrospective observational studies based on administrative databases, which were subject to residual confounding, inadequate coding, and misclassification bias. Second, there was considerable variation in the definitions of IDD and the ascertainment of outcomes used across studies. Third, the majority of evidence came from high-income countries and, as such, is not generalizable to low- and middle-income countries (LMICs). Fourth, a few outcomes, such as maternal mortality and neonatal death, were reported on in only a small number of studies. Lastly, there was a lack of data on the severity of disability, social supports, involvement of caregivers, and accessibility of health services. 

Despite limitations, our review has key strengths. It is one of the most extensive syntheses of maternal, obstetric, neonatal and social outcomes of women with IDD that we are aware of. The size of population studies enabled the assessment of common and rare outcomes, such as severe maternal morbidity and death. Moreover, the stability of the results in different health care systems increases the reliability and external validity of the reported relationships.

Future Implications

Future studies should focus on prospective cohort studies to explore mechanisms of adverse pregnancy outcomes in women with IDD and to identify modifiable risk factors. There is a need for standardisation of the definition of IDD and for outcome measures to make it more comparable across studies. Further studies are needed to assess models of disability-inclusive prenatal care, multi-speciality maternity service care, breastfeeding support services, and postpartum interventions that are specifically designed for women with IDD. More diverse representation from LMICs is also needed to capture the diversity across the globe and to inform fair maternal health policies. Finally, women with IDD have significantly higher risks for adverse maternal, obstetric, perinatal, and neonatal outcomes than women without IDD. 

## Conclusions

Hence, women with intellectual and developmental impairments (IDD) are much more likely than women without IDD to experience unfavorable maternal, obstetric, neonatal, and baby outcomes, according to this systematic study. Maternal IDD was consistently linked to higher risks of preterm birth, low birth weight, small-for-gestational-age infants, NICU admission, severe maternal morbidity, stillbirth, neonatal and infant mortality, and other pregnancy-related complications in over 8.4 million pregnancies. Inadequate prenatal care, delayed breastfeeding start, increased smoking throughout pregnancy, and higher socioeconomic disadvantage were among the significant healthcare and social injustices encountered by women with IDD. These results emphasize the necessity of patient-centered, multidisciplinary, disability-inclusive maternity care that takes into account socioeconomic and medical determinants of health. Reducing these discrepancies and improving outcomes may be possible with improved provider education, supportive healthcare policies, and access to specialized prenatal, intrapartum, and postpartum therapies. To improve the knowledge base and direct equitable maternity healthcare for women with IDD, more prospective studies and intervention-based research are necessary, especially from low- and middle-income nations.
